# Cefuroxime, levofloxacin, esomeprazole, and bismuth as first-line therapy for eradicating *Helicobacter pylori* in patients allergic to penicillin

**DOI:** 10.1186/s12876-019-1056-3

**Published:** 2019-07-25

**Authors:** Zhiqiang Song, Wei Fu, Liya Zhou

**Affiliations:** Department of Gastroenterology, Peking University Third Hospital, Beijing, 100191 China

**Keywords:** *Helicobacter pylori*, Penicillin allergy, Cefuroxime, Levofloxacin, Eradication, Safety, Compliance

## Abstract

**Background:**

Eradicating *Helicobacter pylori* infection is clinically challenging, notably in cases with penicillin allergy. Cephalosporin could be used in lieu of amoxicillin to eradicate *Helicobacter pylori*. The current work aimed to assess therapeutic efficacy and safety of a cefuroxime-based quadruple regimen in treatment-naïve individuals with penicillin allergy, as well as patient compliance.

**Methods:**

In the present prospective single-center cohort study, 152 *Helicobacter pylori* infected individuals with penicillin allergy received eradication therapy with cefuroxime (500 mg twice/day), levofloxacin (500 mg once/day), esomeprazole (20 mg twice/day) and bismuth potassium citrate (220 mg twice/day; 14 days). Safety and compliance were evaluated 1 to 3 days upon eradication. The urea breath test was carried out 8 to 12 weeks upon eradication for efficacy assessment.

**Results:**

This quadruple antimicrobial regimen eradicated the pathogen at 85.5% (95% confidence interval (CI) 79.6–90.8%), 88.4% (95% CI 83.0–93.2%) and 90.1% (95% CI 85.2–94.4%) in intention-to-treat, modified intention-to-treat and per-protocol analyses, respectively, with resistance rates of 4.6 and 40.0% in the background of cefuroxime and levofloxacin, respectively. Meanwhile, 21.3% of patients had adverse reactions, but none was serious. A total of 95.3% of patients showed good compliance. Poor compliance and cefuroxime resistance were detected by uni- or multivariate analyses as independent factors predicting therapeutic failure. Eradication rates in patients with dual levofloxacin and cefuroxime susceptibility, isolated levofloxacin resistance, isolated cefuroxime resistance and dual resistance were 97.2, 84.0, 50.0, and 0%, respectively (*P* = 0.002).

**Conclusions:**

Cefuroxime, levofloxacin, esomeprazole, and bismuth achieved decent efficacy, safety and compliance as first-line antimicrobial regimen in patients with *Helicobacter pylori* and penicillin allergy.

## Background

*Helicobacter pylori* (*H. pylori*) infection and associated diseases are important global health problems. Eradication therapy is important in treating and preventing *H. pylori* infection-related diseases [1]. In recent years, *H. pylori* eradication has been increasingly difficult, mostly due to increased antibiotic resistance (e.g.*,* to clarithromycin and metronidazole) and the limited availability of some antibiotics (e.g.*,* tetracycline and furazolidone) in clinical practice [1–4]. Amoxicillin (semi-synthetic penicillin) is the core medicine used for *H. pylori* eradication due to its long-term low resistance rate [2–5]. However, approximately 5–10% of patients cannot take amoxicillin because of penicillin allergy, leading to more difficult *H. pylori* eradication in such cases [6–8]. According to the guidelines of Maastricht V Consensus Report, for patients allergic to penicillin, the proton pump inhibitor (PPI)-clarithromycin-metronidazole triple therapy is recommended as first-line eradication regimen in regions with low resistance to clarithromycin; in geographical areas with elevated resistance to clarithromycin, bismuth quadruple antimicrobial regimen is recommended [1]. Because of generally increased resistance to clarithromycin and metronidazole, PPI-clarithromycin-metronidazole therapy achieves unsatisfactory cure rates in most regions of the world [9–12]. In addition, due to a number of shortcomings (e.g.*,* complicated administration, common adverse reactions, and tetracycline unavailability in many regions), the clinical application of bismuth quadruple therapy has been restricted [13–15]. Therefore, for penicillin-allergic patients, safe, effective and accessible regimens for *H. pylori* eradication are still lacking.

Cephalosporin and penicillin are β-lactam antibiotics, which share the same bactericidal mechanism (competitive inhibition of trans-peptidase, hindering peptidoglycan synthesis to suppress the biological functions or cause cell wall destruction in *H. pylori*) [16–18]. Antimicrobial sensitivity testing in vitro revealed a low resistance rate for cephalosporin in *H. pylori* (similar to that of amoxicillin) [19–21]. The limited data from a few clinical studies demonstrated that cephalosporin-containing regimens achieve relatively satisfactory eradication rates [20, 22, 23]. .These findings suggested that cephalosporin has a high potential for *H. pylori* treatment. On the other hand, early observational studies have shown that approximately 10% of penicillin-allergic patients are also allergic to cephalosporin (cross-allergic phenomena) [24, 25]. However, with the accumulation of treatment experiences and further related researches, it is currently considered that the actual incidence of cross-allergy phenomena is very low [6, 8]. Meanwhile, cross-allergy phenomena appear only in first-generation and some second-generation cephalosporins with the similar side-chain structure of penicillin; other cephalosporins could be safely applied in penicillin-allergic patients [6, 8, 16, 18]. Moreover, cephalosporins are generally characterized by good safety and tolerance, as well as convenience and wide clinical application [6, 8, 22, 23]. In view of these characteristics and advantages, it is reasonable to hypothesize that for penicillin-allergic patients, second or third generation cephalosporins without the penicillin-like side-chain structure could be potential effective alternatives to amoxicillin in eradicating *H. pylori*. To date, no related clinical study in this field has been reported.

Levofloxacin is usually used for rescue eradication of *H. pylori* infection [1, 3]. However, recently published studies indicated that 14-day first-line treatment with amoxicillin-levofloxacin-PPI-bismuth still achieves satisfactory eradication rates in regions with high levofloxacin resistance [20, 26]. This finding suggested that bismuth and levofloxacin have synergistic bactericidal effects. Bismuth increases the sensitivity of *H. pylori* to levofloxacin and, to a certain extent, facilitates the overcoming of resistance to the latter, which results in further improvement of the eradication efficacy [14, 26]. Moreover, our previous study showed that 14-day cefuroxime-levofloxacin-PPI-bismuth treatment results in a relatively good eradication rate (89.8% in the per-protocol analysis) in patients without penicillin allergy [20]. Currently, clinical studies assessing levofloxacin-containing regimens for first-line *H. pylori* eradication in patients with penicillin allergy are unavailable.

Therefore, this work primarily aimed to assess the eradication efficacy of cefuroxime, levofloxacin, esomeprazole, and bismuth (CLEB) regimen in patients infected by *H. pylori* with penicillin allergy. We also evaluated safety and compliance for the above regimen, and analyzed risk factors that affect its efficacy in eradicating *H. pylori*.

## Methods

### Patients and setting

The current prospective cohort study was performed at the Gastroenterology clinic in a tertiary hospital of Beijing, China, from January 2015 to March 2017. Adult patients with dyspepsia and penicillin allergy were enrolled with clinically diagnosed *H. pylori* infection and no prior eradication treatment.

Exclusion criteria were: age < 18 years; medicines with potential to affect results, including PPIs, H_2_-receptor blockers, bismuth salts, and antibiotics within 4 weeks; gastrointestinal cancer; a history of gastric or esophageal operation; severe concomitant disease; known allergy to any study drug; pregnancy or lactation in women.

### Ethical consideration

Each patient provided signed informed consent. This trial had approval from the Ethics Committee of Peking University Third Hospital, Beijing, China, and conformed to the Declaration of Helsinki following Good Clinical Practice. All authors evaluated study results and approved the final manuscript.

### Study procedures

Health care professionals at the Gastroenterology Unit provided a comprehensive explanation regarding the regimen and possible deleterious effects to the included patients. In addition, the patients were instructed orally and in writing regarding the importance of regular mediation intake, with recommendation to continue treatment even in case of mild or moderate adverse reactions and to call physicians for severe secondary effects. They were required to return within 3 days of eradication for the assessment of treatment compliance and adverse events. *H. pylori* eradication was evaluated 8–12 weeks upon treatment by the urea breath test (UBT; UCBT Kit, Atom High Tech, China). This was an open label trial.

Adverse events were determined by asking open-ended questions using patient self-reports and physical examinations, and grouped into the mild (no interference with daily routine), moderate (limited effects on daily routine), severe (marked effects on daily routine and medication discontinuation), and serious (death, hospitalization, disability, or required intervention for permanent damage prevention) types.

Compliance assessed by pill count was considered good (≥ 80% of pills taken) or poor (< 80% of drugs taken). Individuals poorly complying were not taken into account in the per-protocol (PP) analysis.

### Intervention

The CLEB regimen consisted of cefuroxime (500 mg twice/day after breakfast and supper), levofloxacin (500 mg once/day after breakfast), esomeprazole (20 mg twice/day before breakfast and supper) and bismuth potassium citrate (220 mg twice/day before breakfast and supper) for 14 days.

#### *H. pylori* detection

Before enrolment, *H. pylori* infection was assessed as follows: (1) positive rapid urease test (RUT; HPUT-H102, San Qiang Bio & Che, China) and histological Warthin-Starry staining, and (2) positive UBT. Post-therapeutic *H. pylori* detection was by UBT 8 to 12 weeks upon eradication treatment. *H. pylori* infection was deemed eradicated with a single negative UBT.

In the patients who underwent upper endoscopy, gastric tissue biopsies obtained from the antrum were assessed by the RUT. In case of positive RUT, 2 biopsy samples from the antrum and corpus, respectively, were submitted to Warthin-Starry staining and *H. pylori* density evaluation. Two other samples from the antrum and corpus, respectively, were cultured for *H. pylori* and assessed for antibacterial sensitivity. Histological index grading was based on the updated Sydney system [27]. *H. pylori* density assessment was as + (sparse and sporadic), ++ (dense) and +++ (aggregated) distributions. The information about antibiotic resistance was employed for the analysis of parameters affecting eradication efficacy, but not for selecting first-line therapeutics. In case of CLEB therapy failure, the information was used to guide drug selection for second-line eradication therapy.

UBT was carried out following overnight fasting. Baseline breath samples were collected by blowing through plastic straws into 20-ml containers, and capsules with 75 mg of ^13^C-urea were provided to the patients with 100 ml water. Then, breath samples were obtained 30 min later. A difference between baseline and 30-min samples exceeding 4.0 parts/1,000 of ^13^CO_2_ as assessed on a gas isotope ratio mass spectrometer (GIRMS ZC-202, Wan Yi Sci& Tech, China) indicated positive results.

#### *H. pylori* culture and antibacterial sensitivity test

*H. pylori* was cultured or obtained from the gastric mucosa, and in vitro antibiotic resistance was evaluated by the Epsilometer test (AB Biodisk, Sweden) [2, 19]. *H. pylori* strains with minimal inhibitory concentrations (μg/ml) of > 0.5, > 0.5, > 1, > 8, > 2, and > 1 were deemed to show resistance to amoxicillin, cefuroxime, clarithromycin, metronidazole, levofloxacin, and tetracycline, respectively [2, 5, 19, 27–30].

### Statistical analysis

The sample size of 138 cases administered the CLEB regimen produced a 95% confidence interval (CI) reflecting the sample proportion ± 5% with an estimated *H. pylori* first-line eradication rate of 90% (in a pilot trial conducted prior to the present investigation, successful eradication was achieved in 27/30 patients). Therefore, ≥152 cased had to be enrolled to compensate for a 10% withdrawal rate.

The primary outcome was eradication rates in the intention-to-treat (ITT; all patients administered at least one drug dose), modified intention-to-treat (mITT; cases administered at least one drug dose and submitted to UBT), and PP (cases with complete adhesion to the trial protocol, except for poorly compliant individuals) analyses. Secondary outcomes included adverse event and compliance rates.

SPSS v18 (SPSS Inc., USA) was employed for all statistical analyses. *P* <  0.05 indicated statistical significance. Categorical variables were presented as percentages or frequencies, and continuous ones as mean ± SD. Eradication rates and 95%CIs were determined. Pearson’s chi-square or Fisher’s exact test was employed to compare categorical variables. Univariate analysis was carried out to determine factors predicting *H. pylori* eradication after ≥1 drug dose with the patient showing an endpoint. Multivariate logistic regression analysis (backward modeling and likelihood ratio) was performed for variables statistically significant in univariate analysis.

Penicillin allergy definition included any of the following criteria: (1) a history of allergic reactions, such as fever, rash, skin itching, and anaphylactic shock after penicillin received by oral administration, muscular injection, and intravenous injection; (2) positive skin test. A total of 20 min after intradermal injection of 0.1 ml of the penicillin skin reagent, the penicillin skin test was considered to be positive with any of the following signs: local skin uplift with red halos, subcutaneous induration with a diameter of more than 1 cm, pseudopodia and itching around the red halos, skin rash all over the body, and anaphylactic shock.

Cigarette smoking was reflected by > 1 cigarette pack/week consumed in the past 6 months. Alcohol drinking was reflected by > 50 g of alcohol/day consumed in the past 6 months. After upper endoscopy, patients showing duodenal and/or gastric ulcers were considered to have peptic ulcer disease, whereas those with no ulcers were regarded as non-ulcer dyspepsia cases. In addition, the cases with dyspepsia not examined by upper endoscopy were considered as having uninvestigated dyspepsia.

## Results

**Fig. 1:**
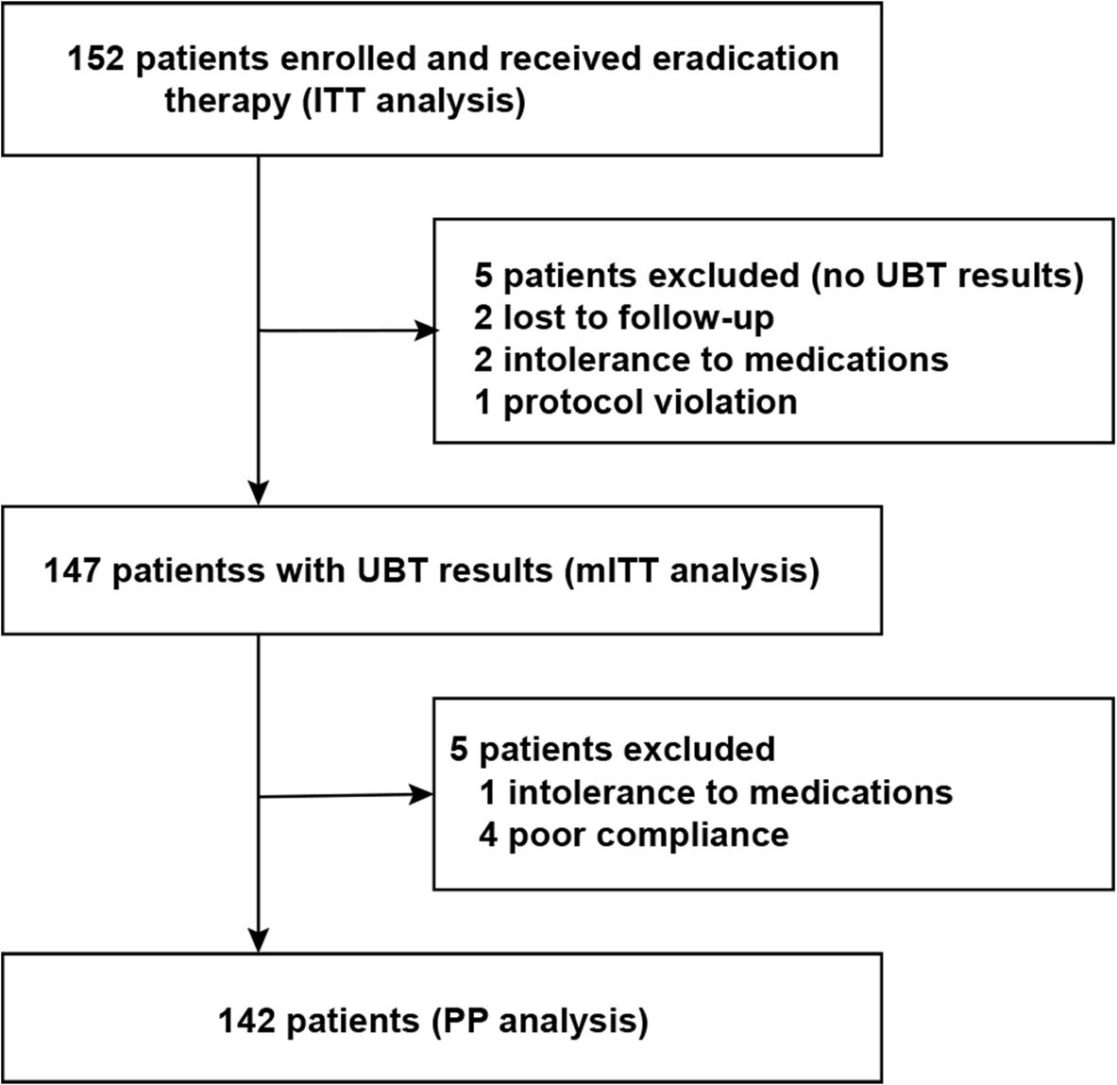
Study flowchart ITT, intention-to-treat; mITT, modified intention-to-treat; PP, per-protocol; UBT, urea breath test

The study flowchart is displayed in Fig. 1. One hundred fifty-two cases were included and administered eradication treatment. Ten cases were excluded from the PP analysis due to loss to follow-up (*n* = 2), intolerance to study drugs (*n* = 3), protocol violation (*n* = 1), and poor compliance (*n* = 4). The baseline properties of all enrolled patients are listed in Table 1. *H. pylori* was detected in 77 patients by upper endoscopy using the RUT and Warthin-Starry staining (these cases underwent *H. pylori* culture, antibacterial sensitivity test, and *H. pylori* density assessment) and 75 patients by the UBT (*H. pylori* culture and antimicrobial sensitivity test were successfully performed in 65 patients or 84.4%).

**Table 1:** Patient baseline features

Variable	Patients enrolled (*n* = 152)
Age, mean ± SD (years)	42.8 ± 13.7
Gender (female/male)	70/82
Body mass index, mean ± SD (kg/m^2^)	23.1 ± 2.8
Cigarette smoking (yes/no)	24/128
Alcohol drinking (yes/no)	28/124
Allergy to penicillin (skin test positive/past allergy history)	112/40
Diagnosis (PUD/NUD/UID)	14/63/75
*H. pylori* density (+/++/+++) ^a^	30/28/19
Amoxicillin resistance (%) ^b^	3.1
Cefuroxime resistance (%) ^b^	4.6
Clarithromycin resistance (%) ^b^	38.5
Metronidazole resistance (%) ^b^	63.1
Levofloxacin resistance (%) ^b^	40.0
Tetracycline resistance (%) ^b^	6.2

*NUD* Non-ulcer dyspepsia, *PUD* Peptic ulcer disease, *UID* Uninvestigated dyspepsia

^a^Data for *H. pylori* density were available in 77 patients

^b^Data for *H. pylori* antibacterial sensitivity were available in 65 patients

### Eradication rates

CLEB treatment yielded eradication rates of 85.5% (95% CI 79.6–90.8%; 130 of 152 patients), 88.4% (95% CI 83.0–93.2%; 130 of 147 patients) and 90.1% (95% CI 85.2–94.4%; 128 of 142) in ITT, mITT and PP analyses, with resistance rates of 4.6 and 40.0% in the background of cefuroxime and levofloxacin, respectively.

### Adverse effects and compliance

A total of 32 (21.3%) patients showed adverse reactions, with 20, 9 and 3 mild, moderate and severe types, respectively. Nevertheless, serious adverse reactions were not observed. Adverse reactions are summarized in Table 2. Good compliance was achieved in 143 (95.3%) cases.

**Table 2:** Adverse events and compliance

Variable, *n* (%)	Eradication therapy (*n* = 150)
Fatigue	18 (12.0)
Anorexia	15 (10.0)
Abdominal pain/discomfort	14 (9.3)
Nausea	12 (8.0)
Diarrhea	12 (8.0)
Headache	5 (3.3)
Vomiting	3 (2.0)
Skin rash	2 (1.3)
Taste distortion	2 (1.3)
Dizziness	2 (1.3)
Patients with adverse reactions	32 (21.3)
Mild adverse reactions	20 (13.3)
Moderate adverse reactions	9 (6.0)
Severe adverse reactions (Medication discontinuation)	3 (2.0)
Good compliance	143 (95.3)

Among the enrolled patients, two (one each for lost to follow-up and protocol violation) were not included in adverse effect and compliance assessments

### Parameters affecting eradication efficacy

In univariable analysis, eradication rate was markedly elevated in cases with good compliance compared with the non-compliance group (90.1% vs. 40.0%, *P* <  0.001). Eradication rate was equally affected by cefuroxime resistance (91.8% vs. 33.3%, *P* = 0.002). Age, gender, body mass index, cigarette smoking, alcohol drinking, diagnoses, *H. pylori* density, and levofloxacin resistance did not significantly affect the eradication rate (Table 3). Multivariable analysis further revealed that poor compliance (odds ratio (OR) = 18.000, 95% CI 1.843–175.775, *P* = 0.013) and cefuroxime resistance (36.000, 2.500–518.371, *P* = 0.008) independently predicted therapy failure.

**Table 3:** Variable analysis of factors affecting eradication efficacy

Variable, *n*/N (%)	Univariable analysis		Multivariable analysis	
	Eradication efficacy (*n* = 147)	*P* value	OR (95% CI)	*P* value
Age				
< 35 years	40/45 (88.9)	0.991		
35–55 years	59/67 (88.1)			
> 55 years	31/35 (88.6)			
Gender:				
female	61/68 (89.7)	0.655		
male	69/79 (87.3)			
Body mass index:				
< 22.0 kg/m^2^	38/42 (90.5)	0.856		
22.0–25.0 kg/m^2^	60/68 (88.2)			
> 25.0 kg/m^2^	32/37 (86.5)			
Cigarette smoking:				
yes	19/22 (86.4)	0.742		
no	111/125 (88.8)			
Alcohol drinking:				
yes	21/25 (84.0)	0.447		
no	109/122 (89.3)			
Diagnosis:				
PUD	14/14 (100.0)	0.361		
NUD	53/61 (86.9)			
UID	63/72 (87.5)			
*H. pylori* density ^a^:				
+	25/29 (86.2)	0.929		
++ +++	24/27 (88.9) 17/19 (89.4)			
Compliance:				
good	128/142 (90.1)	< 0.001	18.000	0.013
poor	2/5 (40.0)		(1.843–175.775)	
Cefuroxime resistance ^b^:				
susceptible	56/61 (91.8)	0.002	36.000	
resistant	1/3 (33.3)		(2.500–518.371)	0.008
Levofloxacin resistance ^b^:				
susceptible	36/38 (94.7)	0.079		
resistant	21/26 (80.8)			

*CI* Confidence interval, *NUD* Non-ulcer dyspepsia, *OR* Odds ratio, *PUD* Peptic ulcer disease, *UID* Uninvestigated dyspepsia

^a^Data for *H. pylori* density were available in 75 patients

^b^Data for *H. pylori* antimicrobial sensitivity were available in 64 patients

### Effects of antibiotic resistance on eradication success

The impacts of cefuroxime and levofloxacin resistance on the success rate based on various probable resistance combinations are shown in Table 4. Eradication rates of cases showing dual cefuroxime and levofloxacin susceptibility, single levofloxacin resistance, isolated cefuroxime resistance, and dual cefuroxime and levofloxacin resistance were 97.2, 84.0, 50.0, and 0%, respectively (*P* = 0.002).

**Table 4:** Effects of cefuroxime and levofloxacin resistance on *Helicobacter pylori* eradication

Variable, *n*/N (%)	Eradication efficacy (*n* = 64)
Cefuroxime susceptible and levofloxacin susceptible Cefuroxime susceptible and levofloxacin resistant Cefuroxime resistant and levofloxacin susceptible Cefuroxime resistant and levofloxacin resistant	35/36 (97.2) 21/25 (84.0) 1/2 (50.0) 0/1 (0)

*P* = 0.002

## Discussion

*H. pylori* represents one of the major human pathogens. About 50% of all humans currently have *H. pylori* infection, with 5–10% of cases combined with penicillin allergy [1, 6–8]. Therefore, cases of *H. pylori* infection and penicillin allergy are relatively common and constitutes an important subgroup in *H. pylori* eradication strategies. Eradicating *H. pylori* in individuals allergic to penicillin is an important medical challenge. According to the recommendations of Maastricht V Consensus Report, PPI-clarithromycin-metronidazole and bismuth quadruple therapies are considered first-line eradication regimens in areas with low and high clarithromycin resistance, respectively [1]. Due to the small number of relevant studies and relatively low quality (Table 5) [9–12, 31–33], the degree of evidence is extremely low and the grade of recommendation is weak in the above guidelines. In addition, due to significantly increased resistance, PPI-clarithromycin-metronidazole therapy could not achieve satisfactory eradication efficacies in most regions of the world [9–12]. The clinical application of bismuth quadruple therapy has also been restricted due to complicated administration, frequent adverse reactions, and tetracycline unavailability in many regions [13–15]. Therefore, more related studies are required to establish a new safe, effective, and widely applied regimen for eradicating *H. pylori.*

**Table 5:** Clinical studies of first-line *Helicobacter pylori* eradication in patients allergic to penicillin

Publication year	Country	Center	Number	Regimen	ITT cure rate	PP cure rate	Adverse reaction rate
2005 [10]	Spain	single	12	PPI + CLA + MET 7 days	58%	64%	17%
2005 [31]	Puerto Rico	single	17	PPI + TET + MET 10 days	84%	84%	unavailable
2006 [32]	Japan	single	5	PPI + TET + MET 7–14 days	80%	100%	unavailable
2010 [11]	Spain	multiple	50	PPI + CLA + MET 7 days	54%	55%	10%
2014 [33]	Japan	single	11	PPI + MET+SIT 7–14 days	100%	100%	64%
2015 [9]	Spain	multiple	112	PPI + CLA + MET 7 days	57%	59%	14%
			50	PPI + TET + MET+BIS 10 days	74%	75%	14%
2017 [12]	Japan	single	10	PPI + CLA + MET 7 days	50%	56%	unavailable
			13	VPZ + CLA + MET 7 days	92%	92%	unavailable
			20	PPI + MET+SIT 7 days	100%	100%	unavailable
			14	VPZ + MET+SIT 7 days	93%	100%	unavailable

*BIS* Bismuth, *CLA* Clarithromycin, *MET* Metronidazole, *ITT* Intention-to-treat, *PP* Per-protocol, *PPI* Proton pump inhibitor, *SIT* Sitafloxacin, *TET* Tetracycline, *VPZ* Vonoprazan

In the present study, the CLEB regimen achieved satisfactory eradication efficacy (85.5, 88.4, and 90.1% in ITT, mITT, and PP analyses, respectively) even in an area with high resistance (38.5% to clarithromycin, 63.1% to metronidazole, and 40.0% to levofloxacin). Moreover, both safety (incidence of side effects of only about 20%) and compliance (good compliance beyond 95%) data were good. The majority of adverse events were transient and mild or moderate, and no overt cross-allergic reactions occurred. These findings provide a very promising new eradication regimen for *H. pylori* infected cases with penicillin allergy.

As mentioned above, cephalosporin could be a good alternative to amoxicillin for *H. pylori* eradication in patients with penicillin allergy. In this study, we used cefuroxime, mainly for the following reasons. (1) Cefuroxime and amoxicillin have an identical bactericidal mechanism through inhibition of cell wall synthesis [16–18]. (2) Cefuroxime is active against *H. pylori* [19, 20]. .The resistance rate in this study was only 4.6%, similar to that of amoxicillin (3.1%). (3) It is widely available in clinical practice. (4) Its safety and tolerance are exceedingly good. (5) Our previous study indicated that the CLEB regimen achieves relatively good eradication efficacy in patients without penicillin allergy, with good safety and compliance [20]; (6) As a second-generation cephalosporin, cefuroxime does not have a side chain structure similar to amoxicillin, showing no overt cross-allergy phenomenon [6, 8, 16, 18]. The present study further confirmed that no obvious cross-allergic effects were observed.

In the present study, another crucial antibiotic was levofloxacin, and the main reasons for its selection are described below. (1) Levofloxacin is broadly available in clinic. (2) Its overall safety and tolerability are good. (3) Recently published clinical studies have shown that 14-day amoxicillin-levofloxacin-PPI-bismuth quadruple antimicrobial treatment as a first-line regimen achieves satisfactory eradication rates even in areas showing elevated levofloxacin resistance [20, 26]. A study further by our group showed that 14-day cefuroxime-levofloxacin-PPI-bismuth quadruple antimicrobial treatment also yields relatively good eradication rates in patients without penicillin allergy [20]. Previous studies revealed that at levofloxacin resistance rates reaching 15–20%, levofloxacin-containing triple regimens do not yield acceptable eradication efficacy [5, 26]. In the current work, however, levofloxacin resistance rate reached 40%, but the CLEB regimen still showed relatively good efficacy, which may be closely related to the inclusion of bismuth. On one hand, bismuth possesses antimicrobial properties itself [14, 34]. On the other hand, bismuth helped overcome *H. pylori* resistance to levofloxacin, further improving eradication efficacy. This might be explained by that bismuth suppresses proton translocation to the bacterial cytoplasm and maintains intracellular pH at a level beneficial for metabolism and division in bacteria, enhancing the antibacterial efficacies of antimicrobials [34, 35].

As shown above, uni- and multi-variable analyses revealed antibiotic resistance and poor compliance as risk factors for failed eradication of *H. pylori* related disease, confirming that antibiotic sensitivity and treatment compliance represent the two top parameters determining successful *H. pylori* treatment [1, 36].

At present, tetracycline is difficult to obtain clinically in the mainland of China, so bismuth-containing quadruple therapy (bismuth, metronidazole, tetracycline and PPI) is rarely used. In the case of penicillin allergy, most patients were received two antibiotics from clarithromycin, levofloxacin and metronidazole for eradication. These three antibiotics have high resistance rates in the mainland of China, and the cure rate is poor and unsatisfactory. According to the relevant expert experiences and past study reports, the eradication rates were estimated only about 50–60% [9–12]. This study provides us a promising regimen for the patients allergic to penicillin. If the CLEB regimen fails, it will be very difficult. Tetracycline, furazolidone and rifabutin are difficult to obtain clinically, and the resistance rates of clarithromycin and metronidazole are very high. For the patients with penicillin allergy in the mainland of China, maybe other options could be chosen for the next eradication, such as minocycline/metronidazole containing quadruple regimen [37] or eradication therapy based on culture and susceptibility.

The present work had limitations: (1) All patients were from the same hospital, and multi-center trials in various world areas are required to confirm the present results. (2) The previous antibiotic use experience would affect the eradication rate, probably related to secondary antibiotic resistance from previous treatment [1, 17]. Unfortunately, in this study we did not ask about the history of antibiotic use for other infectious diseases. In the future research, we can do an in-depth and accurate evaluation and discussion on this topic. (3) In addition, no control group was set. Therefore, it remains unknown whether any actual differences exist in eradication efficacy, safety, and compliance between this regimen and other recommended regimens for patents with penicillin allergy. Nevertheless, the present study provides an important reference for carrying out further related studies.

## Conclusions

Overall, quadruple therapy with cefuroxime, levofloxacin, esomeprazole and bismuth achieves satisfactory eradication effectiveness, safety and compliance as first-line treatment in *H. pylori* infected cases with penicillin allergy.

## Data Availability

The datasets employed and/or analyzed in this study are available from the corresponding author upon reasonable request.

## Abbreviations

*H. pylori*: *Helicobacter pylori*
PP: Per-protocol
PPI: Proton pump inhibitor
